# Can e-learning help you to connect compassionately? Commentary on a palliative care e-learning resource for India

**DOI:** 10.3332/ecancer.2017.ed72

**Published:** 2017-10-19

**Authors:** Soumitra Shankar Datta, Sanjit Agrawal

**Affiliations:** 1 Department of Palliative Care and Psycho-oncology, Tata Medical Centre, Kolkata, West Bengal, India 700160; 2 UCL EGA Institute for Women’s Health (IfWH), UCL School of Life and Medical Sciences, University College London, UK; 3 Department of Breast Surgery, Tata Medical Centre, Kolkata, West Bengal, India 700160

**Keywords:** palliative, e-learning, compassion, India

## Abstract

e-learning resources need to be customised to the audience and learners to make them culturally relevant. The ‘*Palliative care e-learning resource for health care professionals in India’* has been developed by the Karunashraya Hospice, Bengaluru in collaboration with the Cardiff Palliative Care Education Team, Wales to address the training needs of professionals in India. The resource, comprising over 20 modules, integrates psychological, social and medical care for patients requiring palliative care for cancer and other diseases. With increased internet usage, it would help in training a large number of professionals and volunteers in India who want to work in the field of palliative care.

## Introduction

With the progress of modern medicine, the outcome of some diseases has significantly improved in the last 20 years in India. However there remains a significant number of people in the country who do not have access to western medicine and good quality health care facilities. The million death study looked at the various causes of mortality in India [1]. Non-communicable diseases like cancer as well as infectious diseases were found to be contributing to the overall mortality. In cancer care, India lags behind many of the developed countries in terms of provision of screening, early diagnosis, cancer specific treatment facilities near to the patient’s home and access to palliative care. The ‘*Palliative care e-learning resource for health care professionals in India’* [2] aspires to reach out to clinicians and volunteers who are interested to learn about palliative care through the *e*cancer initiative. The e-learning course is spread over 20 modules and covers a wide range of topics such as Basic Principles of Palliative Care, Communication and Counselling Skills, Community Care and Teamwork, End of Life Care, Grief and Bereavement, Opioids, Palliative Care in Non-malignant Disease, Psychosocial Care, Spiritual Care and a few modules on symptom management. Each module has an introduction, a few slides highlighting the key ‘take-home’ points, videos of experts talking on the topic which is covered by that module and also a quiz at the end.

## Connecting compassionately after learning in the virtual space

In the day to day practice of palliative medicine, it is vitally important to connect to the person beyond the body, in a humane and compassionate way. The obvious question that comes to mind is if learners are empowered and encouraged to be ‘compassionate’ after participating in an e-learning course. Recently, audiovisual materials for an online ‘caring curriculum’ were developed for nursing staff to work in a more thoughtful and caring way and this was tested in a controlled experimental design which showed that the staff in the intervention arm had more frequent caring behavior as reported by the patients as opposed to the control arm who did not participate in the course [3]. There are encouraging published personal accounts of using various virtual platforms to improve skills [4]. Globally there are many online courses for developing skills in palliative care but there is very little information on the process of developing an effective and useful course [5]. Finer areas on customising online resources have been commented upon and some authors believe in individualizing the course content as per the need of the learner [6]. Sharing of personal narratives has been shown to improve the reflective capabilities of the learner [7].

## Scope of e-learning in India

The Internet and Mobile Association of India (IMAI), in partnership with IMRB International, published a report in 2016 that mentioned that Urban India has close to 60% Internet penetration and in rural India there are a potential 750 million internet users [8]. Internet use is increasing at a rapid pace in India. However, unlike the west, most Indians are better at downloading or gathering information from the internet and do not necessarily interact with websites in any other way. Given the increasing trend of internet use, developing an e-learning resource on palliative care was timely and appropriate. Learning could be auditory, visual or multimodal and the e-learning modules try to engage all the senses of the learner in order to optimise knowledge and skills transfer.

## e-learning options available to people interested in palliative care in India

There are several e-learning options available to health professionals in India wanting to learn about the practice of palliative medicine as summarised in Table 1. Many of these courses are very well designed and accessed by clinicians around the world. However, there are specific cultural nuances and service related issues in India that may not match well-resourced western societies. The two existing e-learning resources with inputs from Indian professionals (that include both in-person hands-on training and distant learning component) are CCEPC (Certificate Course in Essentials of Palliative Care) by the Indian Association of Palliative Care (IAPC) [9] and Project ECHO (Extension for Community Health Outcome) supported modules on managing pain and related symptoms run by the Trivandrum Institute of Palliative Service (TIPS) [10].

The CCEPC course focus on the basics of palliative care and trains medical and paramedical staff. The candidate has to pay a nominal fee to enrol into this course. CCEPC is delivered through 27 different centers in India and is the most popular e-learning resource on palliation in India. This course offers a 2-month distance-learning program with 15 hours of didactic lecture through a face-to-face contact session. Since 2008 more than 5000 students have been trained by this course. The Trivandrum Institute of Palliative Service (TIPS) was one of the first institutes to offer palliative care education in India. The online course of TIPS focuses on pain management and other related aspects of palliation. TIPS was supported by Project ECHO which is an initiative of the University of New Mexico, USA and now provides an online platform to train professionals across the world on various diseases including pain management in India. Following a “hub-and-spoke” design, 18 centers from India, Bhutan and Bangladesh are now connected to TIPS virtually in real time for didactic sessions and to train professionals. The third and youngest initiative ‘*Palliative care e-learning resource for health care professionals in India’* is an online only resource available to volunteers and other front line health care workers. It is reasonable to expect that over time it will be a useful addition to the available resources in India.

## Integrating physical and psychological care

Palliative care is an ‘approach to patient care’ as well as a discipline that has integrated physical aspects of care and psycho-social care. The ‘*Palliative care e-learning resource for health care professionals in India’* makes an attempt to train learners on all aspects of care. The modules cover symptomatic care like cancer pain hand-in-hand with separate modules on psycho-social care, spiritual care, bereavement and other relevant topics. Experts speak on each topic in an easy and accessible way without jargon. Some of the material is emotionally moving and helps the audience to connect with the emotional pain of patients and their family members. This is extremely relevant as modern medicine often makes the practitioners believe that one needs specialists to deliver each part of the care, the dividing line being between the mind and the body.

## Barriers to e-learning in India and potential solutions

Preliminary evidence suggests that e-learning may be as effective as conventional methods of learning for undergraduate medical students for some areas of learning [11]. However for patients and informal caregivers, the evidence base for continued participation in e-learning and the actual impact on patient outcome has not always been established internationally [12]. Internet coverage in India has improved but can be unreliable in remote and rural areas. Being an online-only course, the current palliative medicine e-learning resource may benefit from having an off-line downloadable DVD version that could serve as a training material during community based teaching sessions in remote areas. Also, the internet can be expensive as compared to the earnings of a young learner and make it unaffordable to some learners. Existing palliative care organisations in India should be encouraged to store e-learning resources in their own libraries or give access to the training material such that it becomes truly free to the end-user. In some situations, tablets loaded with the course material can be used in rural areas without internet access. With more affordable smart tablets, this will soon be a reality for many e-learning materials. The IMAI-IMRB International report mentioned that 77% of urban users and 92% of rural users of the internet in India consider mobile to be the primary device for accessing the Internet. e-learning courses should thus be compatible to smart phones and tablets. It may be necessary to have mobile applications as well. With a diverse population who speak different languages, having Hindi and regional language versions of the course material would also help in training non-English speaking learners. We feel that with improving digital literacy in India, the e-learning courses may be of value to deliver knowledge across boundaries and international borders. The ‘*Palliative care e-learning resource for health care professionals in India’* is a step forward towards the correct direction.

## Conclusion

Future e-learning programmes on palliative care for India may think of having some live webinars, active online groups providing peer support and feedback. Actual patient videos will be extremely valuable if this can be done with appropriate informed consent. India is probably ready for e-learning and we need to study if effective skill transfer happens after a participant completes the course. This may require a systematic randomised trial to evaluate all aspects of the learning experience. If e-learning modules help to disseminate knowledge and skills needed to deliver palliative care delivery in India, it will be a significant boon to many patients and their family members.

## Conflicts of interest

None

## Figures and Tables

**Table 1.** International online course available to health care workers globally including India.

Course name	Organization	Country	Brief description
Macmillan Out of Hours Palliative Care e-learning Course	Macmillan Cancer Support	United Kingdom	Launched in 2011, online e module for learning palliative care for general practitioner. http://learnzone.org.uk/courses/course.php?id=35
Training in end of life care – e ELCA	E Integrity	United Kingdom	*e-ELCA* features over 150 highly interactive sessions that are arranged into eight modules. Some of the modules are publicly available. https://www.e-lfh.org.uk/programmes/end-of-life-care-for-all-public-access/
ICPCN’s e-learning programme	International Children’s Palliative Care Network	South Africa and United Kingdom	e-learning course on all aspects of children’s palliative care available in multiple languages. http://www.icpcn.org/icpcns-e-learning-programme/
Palliative Care: Getting Started	Centre for Palliative Care (the Centre)	United Kingdom	These modules are designed to be introducing principles of palliative care for any health care professional. https://www.centreforpallcare.org/page/31/online-courses
Palliative Care E-OL	National Hospice and Palliative Care Organization (NHPCO)	United States	The multiple course modules target health care workers across the board but requires individual or institutional membership. https://www.nhpco.org/online-learning/palliative-care-e-ol
Taking Ownership: Online Learning Module	Pallium - Canada	Canada	These modules train health care professionals from different disciplines on palliative care as part of their daily work. https://www.caresearch.com.au/caresearch/tabid/3618/Default.aspx

## References

[1] Rajesh D, Gupta PC, Ramasundarahettige C (2012). Cancer mortality in India: a nationally representative survey. Lancet.

[2] ecancer education Palliative care e-learning resource for health care professionals in India. http://ecancer.org/education/course/16-palliative-care-e-learning-course-for-healthcare-professionals-in-india.php.

[3] Hsu TC, Chiang-Hanisko L, Lee-Hsieh J (2015). Effectiveness of an online caring curriculum in enhancing nurses’ caring behavior. J Contin Educ Nurs.

[4] Ménage D (2015). Connecting for compassion. Pract Midwife.

[5] Taroco ALC, Valente TCO, Carbogim CS (2017). Distance learning for updating health professionals in palliative care: a systematic review. BMJ Support Palliat Care.

[6] Mastel-Smith B, Post J, Lake P (2015). Online teaching: “are you there, and do you care?”. J Nurs Educ.

[7] Duke P, Grosseman S, Novack DH (2015). Preserving third year medical students’ empathy and enhancing self-reflection using small group “virtual hangout” technology. Med Teach.

[8] The Internet and Mobile Association of India (IAMAI) Mobile Internet in India 2016. http://www.iamai.in/research/reports_details/4860.

[9] Velayudhan Y, Ollapally M, Upadhyaya V (2004). Introduction of palliative care into undergraduate medical and nursing education in India A critical evaluation. Indian J Palliat Care.

[10] Extension for Community Health Outcome (ECHO) Multimodal approach to pain management. http://palliumindia.org/courses/echo/.

[11] George PP, Papachristou N, Belisario JM (2014). Online e-learning for undergraduates in health professions: a systematic review of the impact on knowledge, skills, attitudes and satisfaction. J Glob Health.

[12] Gustafson DH, DuBenske LL, Namkoong (2013). An eHealth system supporting palliative care for patients with nonsmall cell lung cancer: a randomized trial. Cancer.

